# “So Many Other Things Improve” with Transdiagnostic Treatment for Sleep and Circadian Problems: Interviews with Community Providers on Treating Clients with Serious Mental Illness

**DOI:** 10.1007/s10488-024-01410-1

**Published:** 2024-09-09

**Authors:** Laurel D. Sarfan, Zia Bajwa, Marlen Diaz, Sondra Tiab, Krista Fisher, Emma R. Agnew, Shayna A. Howlett, Sophia Oliver, Catherine A. Callaway, Allison G. Harvey

**Affiliations:** 1 Department of Psychology, University of California, Berkeley, 2121 Berkeley Way, Berkeley, CA 94720-1650 USA; 2 Department of Research & Evaluation, Kaiser Permanente Southern California, Pasadena, CA USA; 3 Department of Psychology, University of California, Davis, Davis, CA USA

**Keywords:** Transdiagnostic, Sleep and Circadian, Serious Mental Illness, Implementation Outcomes, Providers, Interviews

## Abstract

Community mental health centers (CMHCs) offer invaluable, publicly-funded treatment for serious mental illness (SMI). Unfortunately, evidence-based psychological treatments are often not delivered at CMHCs, in part due to implementation barriers, such as limited time, high caseloads, and complex clinical presentations. Transdiagnostic treatments may help address these barriers, because they allow providers to treat symptoms across multiple disorders concurrently. However, little research has investigated CMHC providers’ experiences of delivering transdiagnostic treatments “on the ground,” particularly for adults with SMI. Thus, the aim of the present study was to assess CMHC providers’ perspectives on delivering a transdiagnostic treatment – the Transdiagnostic Intervention for Sleep and Circadian Dysfunction (TranS-C) – to adults diagnosed with SMI. In the context of a larger parent trial, providers were randomized to deliver a standard version of TranS-C (Standard TranS-C) or a version adapted to the CMHC context (Adapted TranS-C). Twenty-five providers from the parent trial participated in a semi-structured interview (*n* = 10 Standard TranS-C; *n* = 15 from Adapted TranS-C). Responses were deductively and inductively coded to identify themes related to Proctor’s taxonomy of implementation outcomes. Four novel “transdiagnostic take homes” were identified: (1) transdiagnostic targets, such as sleep, can be perceived as motivating and appropriate when treating SMI, (2) strategies to bolster client motivation/adherence and address a wider range of symptom severity may improve transdiagnostic treatments, (3) balancing feasibility with offering in-depth resources is an important challenge for transdiagnostic treatment development, and (4) adapting transdiagnostic treatments to the CMHC context may improve provider perceptions of implementation outcomes.

**Supplementary Information:**

The online version contains supplementary material available at 10.1007/s10488-024-01410-1.

## Introduction

Serious mental illness (SMI) affects more than 5% of adults in the United States (National Institute of Mental Health, 2022). According to the National Institute of Mental Health (2022), SMI can be operationalized as one or more psychiatric disorders (e.g., schizophrenia, bipolar disorder, major depressive disorder, posttraumatic stress disorder) that substantially interferes with or limits at least one major life activity (National Institute of Mental Health, 2022). When compared to the general population, people with SMI have lower quality of life, more chronic health conditions, and shorter life expectancy (Baughman et al., 2016; Chesney et al., 2014; Evans et al., 2007). In the United States, community mental health centers (CMHCs) offer publicly-funded services, including treatment for individuals diagnosed with SMI (Drake & Latimer, 2012). Although these publicly-funded services are a vital source of care for people with SMI, there are many barriers to the implementation of evidence-based psychological treatments (EBPTs), such as lack of funding, limited time, and the size and complexity of providers’ workloads (e.g., Aarons et al., 2009; Becker-Haimes et al., 2017; Belling et al., 2011; Brennan et al., 2022; Finley et al., 2015; Greenwood et al., 2000).

One potential solution to help overcome these barriers is *transdiagnostic* EBPTs. In contrast to EBPTs that focus on a single disorder (e.g., depression, insomnia), transdiagnostic EBPTs aim to treat multiple disorders by addressing processes or mechanisms that are common across disorders (Fairburn et al., 2003; Harvey et al., 2004). Transdiagnostic treatments may have advantages in CMHC settings. For instance, they may allow providers to treat many clients with complex diagnostic profiles using a single treatment, which could reduce time needed for training, supervision, and session preparation. Emerging research suggests that transdiagnostic EBPTs can be effective in community settings (e.g., Bolton et al., 2014; Harvey et al., 2021; Liddle, 2016) and are acceptable to clients (e.g., Huynh et al., 2022). Importantly however, few studies have assessed *provider perceptions* of transdiagnostic EBPTs, particularly for providers who specialize in treating adult clients with SMI. This is critical, because providers are vital to the day-to-day delivery of clinical services, and their attitudes and perceptions predict implementation outcomes (e.g., Williams & Beidas, 2019). To our knowledge, only one prior study has reported on interviews with two community providers after having utilized a transdiagnostic treatment for adult clients with SMI (Sauer-Zavala et al., 2019). The findings suggested that providers perceived the treatment—the Unified Protocol (Barlow et al., 2018)—to be acceptable. The present study sought to build on this foundational prior work by interviewing a larger sample of providers regarding a range of implementation outcomes to help answer the question: how do providers perceive transdiagnostic treatments, when using them ‘on the ground’ in CMHCs with adults diagnosed with SMI?

To this end, for the present study, semi-structured interviews were conducted with CMHC providers. These CMHC providers were recruited from a sample participating in a larger parent trial, funded by the National Institute of Mental Health (R01MH120147). In the parent trial, CMHC providers across ten counties in California, United States were trained via facilitation to deliver a transdiagnostic treatment, called the Transdiagnostic Intervention for Sleep and Circadian Dysfunction (TranS-C) to clients with SMI (Harvey & Buysse, 2017).

Based on the Sleep Health Framework (Buysse, 2014), TranS-C was developed in response to evidence that (a) sleep and circadian problems predict and predate SMI diagnoses (Baglioni et al., 2011; Dolsen et al., 2014; Hertenstein et al., 2019; Kaplan et al., 2015; Kivelä et al., 2018; Vargas et al., 2019), and (b) improving sleep and circadian problems can improve SMI (Asarnow et al., 2013; Harvey et al., 2021; Scott et al., 2021; Taylor & Pruiksma, 2014). TranS-C is transdiagnostic in two ways: it is designed to treat multiple SMI diagnoses by addressing multiple sleep and circadian problems (e.g., insufficient sleep, hypersomnia, nightmares, delayed circadian phase) that can maintain and predict SMI (Asarnow et al., 2013; Hertenstein et al., 2019; Kaplan et al., 2015; Slavish et al., 2022).

In a prior randomized controlled trial of TranS-C relative to usual care, TranS-C was associated with medium- to large-sized improvements in all primary outcomes from pre- to post-treatment (i.e., overall impairment *d* = -0.58; psychiatric symptoms *d* = -0.64; and sleep and circadian problems *d* = -0.81 to -0.96) when delivered to adult clients with SMI, who were recruited from and treated in a CMHC. All effects were sustained at six-month follow-up except overall impairment (although effects were in the hypothesized direction, *d* = -0.37). In this prior study, usual care was delivered by CMHC providers, and TranS-C was delivered by masters’ level therapists who (a) were hired and trained within the university affiliated with the study and (b) traveled to the CMHC to deliver TranS-C (Harvey et al., 2021). In a pre-implementation process evaluation for the prior trial, CMHC staff were interviewed to identify potential barriers and facilitators to implementing TranS-C. Among the themes identified, staff reported a need to adapt treatments, including TranS-C, to the CMHC context (Gumport et al., 2020). This feedback, along with theory and pilot data (see Method and Sarfan et al., 2023 for more details), was used to develop an adapted version of TranS-C. In the parent trial of the present study, this adapted version of TranS-C (i.e., Adapted TranS-C) will be compared against the standard version of TranS-C (i.e., Standard TranS-C) in a hybrid type 2 effectiveness-implementation cluster randomized controlled trial, in which CMHCs – and participating CMHC providers therein – were cluster randomized to Standard or Adapted TranS-C (see Sarfan et al., 2023 for protocol and Method for more details).

Although the prior process evaluation yielded important information about implementing TranS-C in CMHCs, a limitation was that CMHC providers’ perceptions of actually *using* TranS-C could not be assessed because, as noted above, (a) the interviews were conducted before implementation, and (b) the providers who delivered TranS-C were hired and trained within a university not the CMHC (Gumport et al., 2020). Moreover, the process evaluation only focused on Standard TranS-C. Indeed, to our knowledge, no prior study has investigated CMHC providers’ perceptions of a transdiagnostic treatment that has been systematically adapted to the CMHC setting.

Using the infrastructure of the parent trial, the present study sought to build on this prior research. The overall aims were to (a) interview *CMHC providers* who had *delivered* TranS-C, and (b) assess their perceptions of *implementation outcomes*, conceptualized according to an established framework, Proctor’s taxonomy of implementation outcomes (Proctor et al., 2011), as well as *influences* on implementation outcomes (Lewis et al., 2018). As an additional aim, we sought to investigate CMHC providers’ perceptions of a typical transdiagnostic treatment (i.e., Standard TranS-C), as well as a transdiagnostic treatment that had been adapted to fit the CMHC context (i.e., Adapted TranS-C).

For additional context, Proctor and colleagues’ (2011) taxonomy identifies and operationalizes several implementation outcomes, including adoption, acceptability, appropriateness, feasibility, fidelity, and sustainability (see Table 1 for definitions) (Proctor et al., 2011). These outcomes can be measured at different levels (e.g., client, provider, leadership). According to Proctor et al. (2011), measuring implementation outcomes from the perspective of partners (e.g., providers, as in the present study) is important for the following three key reasons. First, implementation outcomes indicate the extent to which an implementation effort was successful. Because providers are integral the day-to-day delivery of clinical services, their perspectives should be central when evaluating whether implementation of a clinical intervention was indeed successful (National Cancer Institute, 2019). Second, providers can shed light on implementation processes, including implementation strategies and aspects of the implemented intervention that were helpful or unhelpful. This feedback can be integrated to improve future implementation and treatment development efforts. Third, providers’ perceptions of implementation outcomes can serve as ‘intermediate outcomes’ for downstream clinical and service outcomes. For instance, if a treatment is not perceived as appropriate or feasible to the providers who are delivering it, they are less likely to utilize it routinely over the long term (e.g., Lau et al., 2021).

**Table 1:** Definition of each implementation outcome

Outcome	Definition^a^
Adoption	Uptake; utilization; initial decision to use
Acceptability	Satisfaction with or liking/disliking various aspects/features of the treatment (e.g., content, complexity, comfort, delivery, credibility, research-backed); relative advantage (i.e., “better” than other approaches)
Appropriateness	Perceived fit; relevance; usefulness (i.e., benefits clients)
Feasibility	Suitability for everyday/regular use; compatibility; practicality
Fidelity	Delivered as prescribed in the original protocol or as intended by the developers; adherent to protocol; prescribed dose delivered; quality of program delivery^b^
Sustainability	Maintenance; continuation; durability; incorporation; integration; institutionalization; sustained use; routinization

Note. ^a^All definitions are based on Proctor et al.’s (2011) taxonomy of implementation outcomes. ^b^Based on this definition, fidelity was operationalized in the present study as follows: adhering to the therapist manual and client workbook; focusing on TranS-C for entire sessions each consecutive week; completing TranS-C in the time prescribed by the protocol (8, 50-minute sessions for Standard TranS-C; 4, 20-minute sessions for Adapted TranS-C); and attending supervision. Responses that aligned with this operationalization were coded as “high fidelity.” Responses that deviated from this operationalization were coded as “low fidelity.”

Together, the study’s goals were to advance the field’s understanding of whether transdiagnostic EBPTs (e.g., TranS-C) are indeed implementable in routine practice settings (e.g., CMHCs) from the perspective of providers delivering them “on the ground,” given the potential advantages of these treatments (e.g., addressing complex, comorbid symptoms with a single treatment). We also sought to gather feedback from providers to assess whether they perceived an *adapted* transdiagnostic EBPT to be an improvement relative to a standard version, and whether additional changes should be made to further improve implementation outcomes. Given the limited research on this topic, qualitative methods were used to elucidate themes that, in turn, might inform future research and hypotheses (National Cancer Institute, 2019).

## Method

### Participants and Procedure

Participants (*N* = 25) were recruited via purposeful sampling from the pool of providers recruited for the parent trial (Palinkas et al., 2015). In the parent trial, the eligibility criteria for providers were (1) employed or able to deliver client-facing services within a participating CMHC, (2) interest in learning and delivering TranS-C, and (3) volunteer and formally consent to participate. CMHCs preferred to determine which types of providers were eligible to receive TranS-C training at each site (e.g., case managers, nurses, psychiatrists), because this aligns with their real-world practice. Eligibility criteria for providers to participate in the present study consisted of being enrolled in the parent trial, being employed by a participating CMHC, and having started TranS-C with at least one client. All providers meeting these criteria at the time of recruitment for this study were contacted about their interest (*N* = 58). All providers who expressed interest gave informed consent and were interviewed (*N* = 25). There were no statistically significant differences between contacted providers who were interviewed versus those who were not interviewed, by treatment condition or any of the demographic/professional characteristics assessed for the present study (all *p*s > 0.28; see Table 1 in Online Resource). The sample size was guided by findings that saturation can be reached with an upper bound of 17 interviews (Hennink & Kaiser, 2022). The study was approved by the University of California (UC), Berkeley Committee for Protection of Human Subjects. The parent trial, including a provider interview, was preregistered on clinicaltrials.gov (NCT04154631).

As noted above, the parent trial was a cluster randomized hybrid type 2 effectiveness-implementation trial, in which CMHCs were cluster randomized to the Standard TranS-C condition (*n* = 5 CMHCs) or Adapted TranS-C condition (*n* = 5 CMHCs) (see below for more on treatment conditions). In the parent trial, Standard and Adapted TranS-C were implemented in CMHCs via the implementation strategy of facilitation (Harvey & Kitson, 2015). Specifically, each CMHC received direct support from a lead facilitator (ERA) as well as a team of trained bachelor’s level facilitators, all of whom were employed by the research team and supervised by the parent trial’s principal investigator (AGH). Facilitators supported implementation of TranS-C through a range of activities, including leading TranS-C trainings and ongoing education (e.g., lunchtime lectures), distributing TranS-C manuals and other educational materials, problem solving administrative and organizational barriers, and offering certification in the sleep treatment, as well as weekly supervision and as-needed consultation.

In the parent trial, providers were recruited from the participating CMHCs via collaborative efforts by the facilitators, research team, and key CMHC leadership. Providers were recruited in two phases: the implementation phase (Sarfan et al., 2023) and the train-the-trainer phase (Callaway et al., 2023). All providers in the present study were recruited during the implementation phase, meaning that they were trained by the UC Berkeley facilitators. Providers were generally recruited through flyers posted in CMHCs, announcements in staff meetings, appointments by leadership, and meetings organized by the facilitators. Data collection for the parent trial began in February 2020, marking the start of CMHC providers’ encounters with facilitators. The implementation phase ended for all sites by December 2022. The interviews for the present study took place between August and November of 2021.

In the present study, *n* = 10 providers were from CMHCs in the Standard condition, and *n* = 15 providers were from CMHCs in the Adapted condition. Participating providers were from the following nine counties: four from Santa Cruz, three from Santa Barbara, three from Contra Costa, four from Monterey, two from Solano, two from Santa Clara, three from Placer, two from Alameda, and two from Kings. In some CMHCs, providers were permitted to receive compensation for participation (*n* = 18), whereas in others they were not, per their employers’ policies (*n* = 7). At the time of the interview, providers had started TranS-C with a range of 1 to 8 clients (Mean (*SD*) = 2.88 (*2.17*)). See Table 2 for demographics and professional characteristics of providers in the sample by treatment condition.

**Table 2:** Provider demographics by treatment condition

Characteristic	Standard TranS-C (*n* = 10)		Adapted TranS-C (*n* = 15)		
	*n*	*%*	*n*	*%*	
Sex					
Female	7	70.00	13	86.67	
Male	3	30.00	0	0.00	
Not Reported	0	0.00	1	6.67	
Missing	0	0.00	1	6.67	
Ethnicity					
Hispanic or Latino	3	42.86	3	20.00	
Not Hispanic or Latino	4	57.14	9	75.00	
Missing	3	30.00	3	20.00	
Race					
Asian	1	10.00	1	6.67	
African American or Black	0	0.00	2	13.33	
Multiracial	0	0.00	1	6.67	
White	6	85.71	11	73.33	
Missing	3	30.00	0	0.00	
Degree Type					
Marriage and Family Therapy	4	40.00	2	13.33	
Psychology	1	10.00	4	26.67	
Social Work	3	33.33	3	20.00	
Occupational Therapy	0	0.00	2	13.33	
Other	1	11.11	2	13.33	
Missing	1	11.11	2	13.33	
Therapeutic Approach^a^					
Client Centered	6	60.00	8	53.33	
CBT	9	90.00	5	33.33	
Psychodynamic	3	30.00	2	13.33	
Other	3	30.00	6	40.00	
Licensure					
Licensed	5	50.00	9	60.00	
Not Licensed	5	50.00	5	33.33	
Missing	0	0.00	1	6.67	
	*Mean*	*SD*	*Mean*	*SD*	
Age	38.57	10.80	40.93	10.44	
Caseload	44.25	30.30	18.39	15.18	
Employment Duration	2.83	2.83	5.77	6.36	
Years Since Degree Earned	6.00	3.34	9.69	6.82	

Note.^a^Some providers endorsed more than one therapeutic approach. CBT = cognitive behavioral therapy. Caseload = number of clients on caseload. Employment duration = length of time employed at current CMHC in years

### Treatment

Standard and Adapted TranS-C were delivered alongside the usual care offered by each CMHC. Usual care consisted of working with a provider (e.g., psychologist, case manager, occupational therapist) who offered direct mental health support and referred out for other services as needed (e.g., housing support, vocational specialists). In general, these providers also referred their clients to the parent trial and delivered TranS-C, but sometimes, clients were matched by the research team or CMHC leadership with a different provider to receive TranS-C (e.g., if their primary provider had not completed the TranS-C training). See Table 3 for a comparison of Standard and Adapted TranS-C. In brief, Standard TranS-C consists of eight, 50-minute weekly sessions. Adapted TranS-C consists of four, 20-minute weekly sessions. As depicted in the table, TranS-C in both the Standard and Adapted conditions consists of *core modules*, *cross-cutting modules*,* and optional modules*. With respect to core modules, Standard TranS-C has four core modules, and Adapted TranS-C has five core modules. The core modules form the building blocks of sleep health (e.g., consistent bedtime/wake-time, daytime functioning) and were designed to be delivered to all clients. With respect to cross-cutting modules, both Standard and Adapted TranS-C have the same four cross-cutting modules. The cross-cutting modules focus on treatment *processes* (e.g., motivational enhancement, functional analysis) and are all woven into each session alongside the treatment content. With respect to optional modules, Standard TranS-C has seven optional modules, and Adapted TranS-C has one optional module. The optional modules focus on specialized issues that can arise for people with sleep and circadian problems and SMI (e.g., nightmares, worry, navigating complicated sleep environments) and are used based on case conceptualization, client goals, and clinical judgement. Optional modules are delivered as subsequent separate sessions in Standard TranS-C or integrated in sessions with core modules as needed in Adapted TranS-C. TranS-C was originally designed to be delivered in standalone sessions (i.e., separately from other treatments that a given client may be receiving), such that each session focuses the content of on a core and/or optional module, with the four cross-cutting modules integrated into each session to facilitate therapeutic processes.

**Table 3:** Comparison of standard TranS-C and adapted TranS-C

	Standard	Adapted
**# Sessions**	8	4
**Length of Sessions**	50 min	20 min
**Cross-Cutting Modules** *same across conditions,*** woven into each session***	4 *(Case Formulation*,* Psychoeducation*,* Behavior Change and Motivation*,* and Goal Setting)*	4 *(Case Formulation*,* Psychoeducation*,* Behavior Change and Motivation*,* and Goal Setting)*
**Core Modules**	4 *(Regular Sleep-Wake Times & Wind-Down routine & Wake-Up Routine*,* Improving Daytime Functioning*,* Unhelpful Beliefs about Sleep*,* Maintaining Your Gains)*	5 *(Regular Sleep-Wake Times*,* Wind-down routine*,* Wake-up Routine*,* Improving Daytime Functioning*,* Maintaining Your Gains)*
**Optional Modules**	7 *(Reducing Sleep-Related Worry*,* Improving Sleep Efficiency*,* Reducing Time in Bed*,* Delayed or Advanced Phase*,* CPAP Machine and Exposure*,* Negotiating Complicated Environments*,* Reducing Nightmares)*	1 *(Reducing Sleep-Related Worry)*
**Training**	6–8 h	4 h

Note. The number of modules does not necessarily add up to the number of sessions for a few reasons. First, a provider can choose which of the optional modules to deliver for a given client. In Standard TranS-C, the optional modules are delivered as separate sessions. In Adapted TranS-C, the optional module is integrated across sessions as needed. Second, the core modules of Regular Sleep-Wake Times, Wind-Down Routine, and Wake-Up Routine are presented as one core module in Standard TranS-C and three separate core modules in Adapted TranS-C. In Adapted TranS-C, Improving Daytime Functioning and Maintaining Your Gains modules are delivered in one session via compressed content. Third, the cross-cutting modules are all woven into each session alongside the content from core/optional modules

Adapted TranS-C was systematically developed from Standard TranS-C to better fit the CMHC context. The adaptation process followed the Enhanced Replicating Effective Programs framework (Kilbourne et al., 2007, 2013) and incorporated theory, data, and stakeholder input. See Sarfan et al. (2023) for a detailed description of the adaptation process. In summary, the adapted version of the treatment was designed to retain the core elements of TranS-C – with respect to the Sleep Health Framework (Buysse, 2014) and most commonly utilized treatment skills in prior trials (unpublished data) – while shortening the treatment to make it more feasible for providers to integrate in their routine practice, per provider feedback from interviews (Gumport et al., 2020) and pilot testing (unpublished data).

### Measures

Provider characteristics were measured using a demographics and work history questionnaire delivered for the parent study. This measure assessed age, sex assigned at birth, ethnicity, race, number of clients on caseload, length of time employed at the provider’s CMHC, type of degree, year completed degree, theoretical orientation, and licensure status.

The primary measure was a semi-structured interview that was developed for the present study based on Proctor’s taxonomy of implementation outcomes (Proctor et al., 2011). Each interview question was constructed to assess one of these implementation outcomes: adoption, acceptability, appropriateness, feasibility, fidelity, and sustainability. See Table 1 for definitions of each outcome. The first author (LDS) devised the first draft of the interview, which was refined with input from the facilitators and members of the research team. See Online Resource (page 13) for each interview question with the corresponding implementation outcome. Note that outcomes of penetration and implementation cost were not assessed by the interview, based on Proctor’s recommendations that these outcomes should be assessed by administrative data, case audit, and/or checklists, rather than a semi-structured interview (Proctor et al., 2011). The interviews were audio recorded and conducted over the phone by the first author (LDS) and a trained project coordinator (ST). Recommended strategies from prior research were used to minimize social desirability bias during the interviews (e.g., developing rapport, providing assurance, prefacing questions, probing and requesting examples) (Bergen & Labonté, 2020).

### Analysis

Interviews were a mean length of 24.77 min (*SD* = 5.56). Each interview was transcribed verbatim using a secure, online service (note that interview recordings did not contain identifying provider or client information). Next, each interview was checked and corrected against the original audio recording by an undergraduate research assistant, then checked again and further corrected by a different undergraduate research assistant.

To analyze the interviews, an iterative process of deductive and inductive analysis was used (Braun & Clarke, 2006; Miles et al., 2019). In the deductive phase, a codebook was developed based on Proctor’s taxonomy of implementation outcomes (Proctor et al., 2011), which included the following five deductive codes: acceptability, appropriateness, fidelity, feasibility, and sustainability. Through weekly meetings and email feedback, a coding team of seven undergraduate research assistants was trained to identify each of these codes in the transcripts, until Cohen’s kappa of 0.80 was achieved between the coders and the team lead (LDS), indicating substantial agreement (Landis & Koch, 1977). Two coders (ZB, SO) from the coding team were selected to continue to the inductive phase based on their high inter-rater reliability with the team lead (Cohen’s kappa = 1.0).

In the inductive phase, the team lead and one of the selected coders (ZB) reread all the transcripts and identified *inductive subcodes* that emerged related to each *deductive code*. In general, these inductive subcodes addressed details and reasons underlying the deductive codes. For example, for the deductive code of acceptability, several inductive subcodes emerged, including “more comprehensive than other approaches,” “advantages over medication,” and “therapist liked the structure.” The team lead and coder met regularly to refine the inductive subcodes, which were compiled into an inductive codebook. Note that the implementation outcome of “adoption” was left out of the deductive codebook due to administrative error; however, inductive subcodes related to this implementation outcome emerged and were included in the inductive codebook. After this generation stage, there were *n* = 262 inductive subcodes, which fell under *n* = 12 overarching *categories* related to the deductive codes. The inductive subcodes were then consolidated by the team lead and coder, such that redundant subcodes were consolidated and imprecise subcodes were removed. This process left *n* = 213 inductive subcodes in the codebook. Next, the team lead and both selected coders reread and inductively coded each transcript according to the inductive codebook. Subcodes were recursively refined, added, and consolidated during this process (e.g., Braun & Clarke, 2006), and discrepancies were resolved by the team lead. By the end of the inductive coding process, the two coders and team lead stopped needing to refine, add, or consolidate subcodes. In other words, no new insights or issues were emerging from the data, supporting thematic saturation (Hennink et al., 2017).

After inductive coding, the team grouped the inductive subcodes into *n* = 76 similar *themes* to facilitate interpretation (Braun & Clarke, 2006). For instance, the subcodes of “more client centered,” “more options to tailor treatment to clients,” “gradual approach for clients,” and “more participatory for clients” were grouped into the theme of “more client centered.” Finally, transcripts were sorted by condition (Standard vs. Adapted TranS-C), and the proportion of providers who endorsed each theme was calculated by condition and for the total sample.

## Results

See Online Resource Table 2 for all themes identified during inductive coding, frequency of each theme by condition and total sample, and representative quote/s for each theme. In the section that follows, the percentages in the parentheses indicate the proportion of providers who had a response coded with a given theme in each condition (i.e., S = proportion of Standard providers, A = proportion of Adapted providers). The results are organized by overarching category. To facilitate readability, *conditions* and the *core idea* of each theme are italicized. When comparisons are drawn, the comparator is always the other condition (e.g., “A greater proportion of Adapted providers” is relative to the proportion of Standard providers).

### Motivation for Adoption

Most providers in *both conditions* were motivated by beliefs around the *importance of treating sleep* (S = 60.0%, A = 86.7%). For instance, providers recognized the impact of sleep on mental health and felt that sleep treatment was relevant to their clients. Additionally, several providers in *both conditions* said that they were motivated to adopt TranS-C, because it represented a *professional development or learning opportunity* (S = 40%, A = 40%) and seemed like a *good fit with their organization’s approach to care or the provider’s professional role* (S = 30.0%, A = 26.7%). Slightly more *Adapted* providers were motivated by *characteristics of TranS-C* (e.g., research-based, structured, achievable for clients) (S = 40%, A = 53.3.%) and their *personal relationship to sleep* (e.g., valuing their own sleep; personal history of sleep problems) (S = 30.0%, A = 46.7%).

### Acceptable

In *both conditions*, most providers identified TranS-C *content* as acceptable (S = 80.0%, A = 80.0%); in particular, providers said that the information was relevant/useful and the manual and workbooks were well-organized and easy to use. Additionally, more than half of providers in *both conditions* said that TranS-C was *more comprehensive* relative to other sleep treatment approaches (S = 60.0%, A = 73.3%). A few providers in *both conditions* said that the treatment seemed *credible*, particularly the research support and affiliation of the research institution (S = 20.0%, A = 20.0%) and conferred *advantages compared to medication* (S = 20.0%, A = 20.0%). A few providers in *both conditions* also said that sleep treatment was *less stigmatizing*, compared to treatments for other mental health problems (S = 10.0%, A = 13.3%), and that TranS-C *complemented other approaches* that they used to treat sleep (S = 10.0%, A = 13.3%). In the *Adapted* condition, more providers reported liking the treatment’s *structure* (S = 10.0%, A = 53.3%) and *flexibility* (S = 0.0%, A = 33.3%) and said that the treatment was *more helpful* (S = 10.0%, A = 33.3%), *client-centered* (S = 0.0%, A = 26.7%), and *better organized* (S = 40.0%, A = 53.3%), relative to other approaches to treat sleep.

### Not Acceptable

More than half of providers in *both conditions* said that TranS-C was *overly simplified* (S = 60.0%, A = 73.3%), reporting that they were interested in more in-depth materials on a wider range of topics (e.g., sleep and psychotic symptoms, anxiety, pain). Slightly more *Adapted* providers said *TranS-C seemed similar* to other approaches used to address sleep (S = 10.0%, A = 20.0%) and that *clients disliked using written materials and worksheets* (S = 10.0%, A = 33.3%). More providers in the *Standard* condition reported that TranS-C was *too fast-paced*, with too much material slotted for each session (S = 60.0%, A = 26.7%), and had *suggestions to improve flexibility* (S = 20.0%, A = 6.7%). Related, one provider in the *Standard* condition reported that the treatment was *too structured* (S = 10.0%, A = 0.0%).

### Appropriate

Most providers in *both conditions* said *sleep is an important topic*, with many providers noting that sleep impacts their clients’ mental health and their clients want to focus on sleep (S = 90.0%, A = 73.3%). Most providers in *both conditions* also said that *TranS-C helps clients* with sleep and mental health (S = 80.0%, A = 73.3%) and is *appropriate for treating sleep issues* (S = 80.0%, A = 73.3%). Additionally, a few providers in *both conditions* felt that TranS-C was appropriate for clients with *different types of symptoms* (e.g., trauma, psychosis, anxiety) (S = 20.0%, A = 20.0%) and felt *empowering* for providers and clients (S = 20.0%, A = 20.0%). More *Adapted* providers said that TranS-C was *beneficial for any clinical presentation*, including for any diagnosis (S = 40.0%, A = 73.3%), as well as across *other client characteristics* (e.g., alliance, consistency) (S = 0.0%, 26.7%). Two *Adapted* providers felt that TranS-C *fit with their professional role* (S = 0.0%, A = 13.3%).

### Not Appropriate

Most providers in *both conditions* said that TranS-C did not always seem like a good fit for certain *characteristics of clients* (S = 80.0%, 86.7%), particularly low motivation, non-adherence, and disorganization. A theme that came up more for *Standard* providers was that TranS-C was not appropriate depending on *client symptoms*, particularly when clients were experiencing acute symptoms such as a psychotic episode or crisis (S = 60.0%, A = 33.3%), and that the fit and utility *depended on each client* (S = 80.0%, A = 33.3%). More *Standard* providers also thought that to achieve optimal fit and benefits of Standard TranS-C, *additional sessions and more detailed resources* would be needed (S = 80.0%, A = 40.0%).

### Feasible

Providers in *both conditions* reported that *implementation characteristics*, particularly the support from facilitators, improved feasibility (S = 30.0%, A = 33.3%). Additionally, a few providers in *both conditions* identified *characteristics of themselves* (e.g., valuing/prioritizing TranS-C) (S = 20.0%, A = 20.0%) and their *context* (e.g., organizational support, manageable workload) (S = 20.0%, A = 20.0%) that improved feasibility. A theme that came up more frequently among *Adapted* providers was that *treatment characteristics* improved feasibility, particularly the number of modules, structure, flexibility, ease of learning and delivery, and alignment with clients’ overarching goals (S = 20.0%, A = 73.3%). One provider in the *Standard* condition said that *client characteristics*, such as interest or willingness, improved feasibility (S = 10.0%, A = 0.0%).

### Not Feasible

Most providers in *both conditions* reported that *treatment characteristics* made TranS-C more difficult to deliver—most notably, insufficient time to deliver TranS-C as designed (S = 70.0%, A = 73.3%). Most providers in *both conditions* also said that *client characteristics and clinical presentation* decreased feasibility, particularly symptom severity and low motivation (S = 80.0%, A = 53.3%). A theme that came up more for *Standard* providers was that *contextual factors* (e.g., caseload, administrative responsibilities) decreased feasibility (S = 40.0%, A = 20.0%).

### High Fidelity

Most providers in *both conditions* said that, when delivering TranS-C, they *used the manual and workbook* (S = 90.0%, A = 73.3%). Slightly more *Adapted* providers reported that they *regularly attended supervision* (S = 0.0%, A = 33.3%), *did not make any changes* to the treatment (S = 0.0%; A = 6.7%), and *dedicated full sessions* to TranS-C (S = 40.0%, A = 53.3%).

### Low Fidelity

Most providers in *both conditions* said they used *more time than allotted* for each topic (S = 90.0%, A = 80.0%) and *integrated TranS-C* into sessions focused on other issues (S = 70.0%, A = 73.3%). A few providers in *both conditions* also reported *inconsistent supervision attendance* (S = 20%, A = 13.3%) and *pausing TranS-C* for a week or more to focus on other treatment goals (S = 10.0%, A = 6.7%). Themes that arose for proportionally more *Standard* providers were that they *integrated outside sleep treatment materials* when delivering TranS-C (S = 60.0%, A = 40.0%), *skipped TranS-C materials* (S = 80.0%, A = 33.3%), and *stopped TranS-C* altogether with certain clients (S = 20.0%, A = 6.7%). In contrast, more *Adapted* providers said that they *did not always adhere to the provider manual and client workbook* (S = 0.0%, A = 40.0%) and *personalized their delivery of TranS-C*, such as tailoring delivery to each client (S = 40.0%, A = 66.7%).

### Influences on Fidelity

Most providers in *both conditions* said that deviations from the TranS-C protocol were usually driven by clients’: *characteristics*,* needs*,* and clinical presentation*, such as adapting the treatment to fit a given clients’ cognitive ability, housing situation, or clinical severity (S = 80.0%, A = 73.3%), and *interest/motivation in TranS-C* (S = 50.0%, A = 60.0%). A theme that arose proportionally more among *Standard* providers was that they deviated from the suggested schedule and timing of TranS-C because *more time* was needed to get through the treatment content, both in terms of number of sessions and the length of each session (S = 50.0%, A = 33.3%). One provider in the *Standard* condition also said that they did not attend the supervision group, due to frequent *rescheduling* by the research team (S = 10.0%, A = 0.0%). Slightly more *Adapted* providers said that their *own characteristics* (e.g., priorities around delivering sleep treatment each week) increased fidelity (S = 0.0%, A = 26.7%).

### Sustainable

Providers in *both conditions* said that they would continue to deliver TranS-C in the future, because it was *helpful for their clients* (S = 60.0%, A = 53.3%), and they wanted to *develop proficiency* in delivering it (S = 10.0%, Adapted = 6.7%). Slightly more *Adapted* providers said that they would continue to deliver TranS-C because of their beliefs in the *importance of treating sleep* (S = 10.0%, A = 26.7%), and TranS-C strategies felt *feasible* for providers and clients (S = 10.0%, A = 20.0%). Additionally, more *Adapted* providers said that TranS-C felt sustainable based on *treatment characteristics* (e.g., structure, organization, format/content, research base) (S = 30.0%, A = 66.7%) and *support during implementation* (e.g., consultation offered by facilitators) (S = 20.0%, A = 46.7%). More *Standard* providers said that they would continue to deliver TranS-C, because it felt *relevant* to their clients (S = 80.0%, A = 20.0%).

### Not Sustainable

A few providers in *both conditions* reported that barriers to sustainability included *treatment characteristics* (e.g., client difficulty keeping track of a manual, organization of the manual/workbook) (S = 20.0%, A = 26.7%) and *context* (e.g., limited time, referrals, and prioritization of sleep treatment by the organization) (S = 20.0%, A = 26.7%). More *Adapted* providers reported that, for continued TranS-C delivery, they would need *additional resources or consultation* on sleep and sleep treatment (S = 30.0%, A = 46.7%) and would likely *make modifications* when using TranS-C in the future, such as integrating only parts of the treatment or combining with other approaches (S = 20.0%, A = 46.7%).

## Discussion

The present study investigated CMHC providers’ perceptions of delivering a transdiagnostic treatment—either a standard version (Standard TranS-C) or a version that had been adapted to the CMHC context (Adapted TranS-C)—to clients diagnosed with SMI and sleep and circadian problems. Responses were deductively and inductively coded to identify themes related to implementation effectiveness, per Proctor’s taxonomy of implementation outcomes (Proctor et al., 2011). Common themes emerged across the two treatment conditions, which may offer insights into CMHC providers’ experiences of delivering transdiagnostic treatments more generally. However, distinct themes also arose between the two conditions that, on the whole, highlighted advantages of adapting transdiagnostic treatments to the CMHC context.

The most prominent theme identified by providers in both conditions underscored the importance of treating sleep as a transdiagnostic target. Most providers felt that their clients had sleep problems, and their clients *wanted* to focus on sleep. Providers also felt that sleep problems may represent a less stigmatized path to behavioral health and that sleep treatment plays an important role in improving their clients’ mental health. As one provider said, “I do see with my clients when they have improved sleep, so many other things improve for them. Their mood, their energy levels, their concentration, their focus.” A related group of themes that emerged prominently across the two conditions was related to TranS-C itself. Providers in both conditions liked the content, regularly used the manual and workbook in session, and felt it was credible, empowering, complemented their current practice, and conferred relative advantages – particularly that it was more comprehensive compared to other sleep treatments. Importantly, providers said that TranS-C seemed appropriate and helpful to address their clients’ mental health and sleep symptoms. One provider said, “It’s been, for several clients, life altering.” Although it is not new that treatments focused on sleep can concurrently reduce symptoms of SMI (e.g., Scott et al., 2021; Taylor & Pruiksma, 2014), to our knowledge, this is the first study indicating that *providers* perceive this approach – namely, using transdiagnostic treatments to address sleep and circadian problems for their clients with SMI – to be motivating and appropriate for their routine clinical practice. Moreover, these results are consistent with the one other study, to our knowledge, to suggest that providers appreciate transdiagnostic treatments for adults with SMI *after* having tried them “on the ground” with their clients (Sauer-Zavala et al., 2019). Given that provider attitudes and perceptions predict implementation outcomes (Williams & Beidas, 2019) and a single transdiagnostic treatment may allow providers to help clients with a wide range of clinical presentations (Fairburn et al., 2003; Harvey et al., 2004), these findings underscore the exciting potential of transdiagnostic treatments in CMHCs.

That said, contradictory findings emerged. First, although providers liked the content and treatment materials, they also wanted access to more in-depth resources on a range of topics. Second, as noted above, more time was needed to deliver TranS-C than allotted, even in the briefer Adapted TranS-C condition. Together, these findings represent a dual challenge of transdiagnostic treatments for SMI—and indeed perhaps a challenge of most EBPTs—namely, how can we adequately equip providers to cover a wide range of topics within a feasible timeframe, while addressing the breadth of a given clients’ needs and priorities? One path forward could be modifying the design of the treatment. For instance, more in-depth content could be added, *but* providers could determine which pieces of this additional content to include using flow charts (e.g., Chorpita & Weisz, 2009; Murray et al., 2014) or via shared decision-making with clients (Langer et al., 2022). From another angle, these contradictory themes seem to reflect a, perhaps unsurprising, tension between different levels of implementation, such as system-level (e.g., session lengths to accommodate large caseloads) and client-level needs (e.g., time needed to learn and integrate treatment content) (Bailey et al., 2021). Such tensions may require a multi-pronged approach at treatment and policy levels (e.g., further iterating treatments to fit client and provider needs while equipping CMHCs with resources to meet these needs).

Another finding from both conditions was providers’ frequent report that client characteristics impacted providers’ fidelity and the perceived appropriateness of TranS-C. With respect to fidelity, providers said that they deviated from the treatment manual/workbook in response to clients’ presentation. These changes are not necessarily problematic; indeed, some findings suggest that provider-initiated ad hoc adaptations that are made to improve fit between treatment and clients may positively impact outcomes (e.g., Aschbrenner et al., 2021). Research from our team is currently underway to test the impact of such adaptations on client outcomes in the parent trial (Diaz et al., under review). With respect to the implementation outcome of appropriateness, providers in both conditions felt that some clients’ limited motivation and compliance curbed the utility and fit of TranS-C. Notably, motivational enhancement is included as a cross-cutting module in both TranS-C treatment conditions. Specifically, in each core and optional module, motivational enhancement strategies are written into the manual and workbook (e.g., clients are asked to make a list of pros and cons). However, the present findings suggest that the motivation-specific content should be strengthened, perhaps via evidence-based strategies like ongoing supervision and coaching that is directly focused on motivational enhancement (Schwalbe et al., 2014).

When looking at differences in themes between the two conditions, an overall pattern emerged: across most categories and themes, more Adapted providers identified strengths of TranS-C, whereas more Standard providers identified areas for improvement. The clearest divergences were observed for themes related to treatment and client characteristics. With respect to treatment characteristics, more Adapted providers identified treatment characteristics that *improved* implementation outcomes, such as the structure, flexibility, and relative benefits of TranS-C compared to other sleep treatments (e.g., more client-centered, organized, and helpful). In contrast, more Standard providers critiqued characteristics of the treatment, saying that it was too fast-paced with too much material, not flexible enough, too structured, and additional sessions with more detailed resources were needed. Similarly, with respect to client characteristics—a critical dimension for transdiagnostic treatments—more Adapted than Standard providers said that the treatment was beneficial across client presentations, diagnoses, and characteristics. In contrast, more Standard than Adapted providers said that the treatment was not always appropriate for clinical symptoms and that the fit/utility depended on each client.

There were exceptions to this overall pattern. For instance, with respect to sustainability, more Standard providers said that they would continue to deliver TranS-C, because it was relevant to clients, whereas more Adapted providers said that they would need additional resources to continue delivering TranS-C. Similarly, although Adapted providers reported that TranS-C was beneficial across clinical presentations, they also reported that client characteristics negatively impacted fidelity and appropriateness, as described above. Future iterations of Adapted TranS-C should integrate this feedback by, for example, adding training, supervision, and modules to help providers address client and treatment characteristics identified as impacting implementation outcomes in the present study (e.g., symptom severity, motivation, client adherence, and housing).

In sum, these collective findings are among the first to suggest that, although improvements may be needed, (a) a transdiagnostic treatment for clients with SMI and (b) that has been systematically adapted to the CMHC setting (e.g., Adapted TranS-C) can result in favorable implementation outcomes – and perhaps even improved outcomes relative to standard transdiagnostic treatments – from the perspective of CMHC providers.

### Limitations and Future Directions

The present findings should be considered in light of limitations. First, although the sample size is comparable to that used in similar qualitative studies (e.g., Bearman et al., 2020; Connors et al., 2019) and providers were interviewed across nine different counties, the sample size may limit transferability of findings. Second, and related, a recent review found that theoretical saturation could be reached with as few as nine interviews (Hennink & Kaiser, 2022). Indeed, by the end of the inductive coding process in the present study, thematic saturation appeared to have been reached. That said, we recognize the complexities of defining and assessing saturation (Hennink et al., 2017) and that different methodological decision points might have influenced this determination (e.g., the coders). Third, of the 58 providers contacted, 25 participated in the interview (43%), which may have introduced response biases. Although (a) interviewed versus contacted non-interviewed providers did not appear to differ on sociodemographic and occupational characteristics assessed for the present study and (b) strategies were used to minimize response bias during the interviews (e.g., developing rapport, providing assurance, prefacing questions, probing and requesting examples) (Bergen & Labonté, 2020), future research should assess whether similar themes emerge across different samples of providers. Fourth, our operationalization of fidelity was weighted toward adherence to and dose of the TranS-C protocols as designed. As a result, the present findings offer limited insights on other dimensions of fidelity, such as the quality of TranS-C delivery (Proctor et al., 2011), and provider adaptations, such as tendencies to make changes that were fidelity consistent versus inconsistent (Wiltsey Stirman et al., 2019). Future research that assesses a wider range of fidelity dimensions might help the field learn more about how providers adapt transdiagnostic treatments in real world settings and the impact of such changes on clinical and implementation outcomes.

To conclude, there may be “transdiagnostic take homes” from this study to inform the development and implementation of transdiagnostic treatments for SMI in CMHCs, as well as ways to improve transdiagnostic treatments that have been adapted to the CMHC setting (e.g., Adapted TranS-C). First, transdiagnostic targets, such as sleep, can be perceived as motivating and appropriate from the perspective of CMHC providers treating clients diagnosed with SMI. Second, adding more training and supervision for providers to address symptom severity, motivational enhancement, and adherence might represent transdiagnostic additions that could improve implementation outcomes. Third, balancing feasibility with in-depth resources for a wide range of client presentations may reflect a key challenge for transdiagnostic treatment development. Fourth, transdiagnostic treatments that have been adapted to the CMHC context may feel more implementable to CMHC providers, relative to standard EBPTs. Future research is needed to empirically evaluate Adapted TranS-C relative to Standard TranS-C (see Sarfan et al., 2023 for planned analyses) and to test approaches to improve feasibility, access to in-depth resources, and client motivation and adherence to transdiagnostic treatments.

## Electronic Supplementary Material

Below is the link to the electronic supplementary material.

Supplementary Material 1

## Declarations

### Guidelines

The present study followed the guidelines set forth by O’Brien et al. (2014)

**Reference for Guidelines*: O’Brien, B., Harris, I., Beckman, T., Reed, D. A., & Cook, D. A. (2014). Standards for reporting qualitative research: A synthesis of recommendations. *Journal of the Association of American Medical Colleges*.
