# Supplementary material for: “So Many Other Things Improve” with Transdiagnostic Treatment for Sleep and Circadian Problems: Interviews with Community Providers on Treating Clients with Serious Mental Illness
Source: Adm Policy Ment Health. 2024 Sep 9;52(2):318–30. doi: 10.1007/s10488-024-01410-1 (PMC11903513; doi:10.1007/s10488-024-01410-1)
Supplement: Supplementary file 1 — Supplementary Material 1 [file 10488_2024_1410_MOESM1_ESM.docx]

**Online Resource: Supplement**

**“So many other things improve” with transdiagnostic treatment for sleep and circadian problems: Interviews with community providers on treating clients with serious mental illness**

Online Resource Table 1. Comparisons Between Providers who were Contacted and Interviewed versus Contacted but not Interviewed by Demographic/Professional Characteristics and Treatment Condition

| **Characteristics** | **χ^2^ *(df)*** | ***p*-value** |
| --- | --- | --- |
| Sex | 1.47 *(2)* | 0.48 |
| Ethnicity | 0.13 *(1)* | 0.72 |
| Race | 6.18 *(5)* | 0.29 |
| Degree Type | 3.64 *(5)* | 0.60 |
| Therapeutic Approach | 15.95 *(17)* | 0.53 |
| Licensure | 0.29 *(1)* | 0.59 |
| TranS-C Condition (Adapted vs. Standard) | 0.03 *(1)* | 0.85 |
|  |  |  |
|  | ***t*-statistic *(df)*** | ***p*-value** |
| Age | 0.28 *(49)* | 0.78 |
| Caseload | 0.24 *(39)* | 0.81 |
| Employment Duration | -0.93 *(53)* | 0.36 |
| Years Since Degree Earned | 0.97 *(45)* | 0.34 |

*Note*. df = degrees of freedom. Chi-square tests were used for categorical variables, and *t*-tests were used for continuous variables.

Online Resource Table 2. Inductive Coding Results

| **Categories** | **Themes** | **Standard**  **N (%)**  **(N = 10)** | **Adapted**  **N (%)**  **(N = 15)** | **Total Sample**  **N (%)**  **(N = 25)** | **Example Quote/s** | |
| --- | --- | --- | --- | --- | --- | --- |
| **Motivation for Adoption** |  |  |  |  |  | |
|  | Personal Relationship to Sleep | 3 (30.0%) | 7 (46.7%) | 10 (40.0%) | "Honestly, I just personally love sleeping." | |
|  | Professional Development and Learning | 4 (40.0%) | 6 (40.0%) | 10 (40.0%) | "Mainly, I'm always wanting to learn more things." "I really liked the idea of having another specialty." | |
|  | Characteristics of Sleep Treatment/Program | 4 (40.0%) | 8 (53.3%) | 12 (48.0%) | "It's research based." "I like structure." | |
|  | Organizational/Professional Role | 3 (30.0%) | 4 (26.7%) | 7 (28.0%) | "My work supported it." "It fit straight in with our, you know, our focus of like hygiene, nutrition, sleeping, getting your laundry done, your grocery shopping done, your budgeting done." | |
|  | Importance of Treating Sleep | 6 (60.0%) | 13 (86.7%) | 19 (76.0%) | "Since I’ve had this job, I can't believe how many clients, you know, they don't sleep well for a few days, and they relapse. They don't sleep well for a few days, and they start having psychotic symptoms they haven’t had in a while." | |
| **Acceptable** |  |  |  |  |  | |
|  | Structure | 1 (10.0%) | 8 (53.3%) | 9 (36.0%) | “We’re going to start working directly from the workbook, because she really needs that structure in helping her create a better sleep routine.” | |
|  | Flexibility | 0 (0.0%) | 5 (33.3%) | 5 (20.0%) | "There was a lot, a lot of flexibility, which I think demonstrates like insight into the process and the reality of like what it's like to work with clients." | |
|  | Content | 8 (80.0%) | 12 (80.0%) | 20 (80.0%) | "There's a lot of useful information." | |
|  | Credibility | 2 (20.0%) | 3 (20.0%) | 5 (20.0%) | “...especially when you put the name UC Berkeley behind it... that really led a lot, lends a lot of credibility for people, and I think it made them more open and willing to listen and hear it.” | |
|  | More Comprehensive | 6 (60.0%) | 11 (73.3%) | 17 (68.0%) | “I like the [UC Berkeley] sleep treatment, because I feels it's flushed out more in terms of how there's different modules, and there's more details and additional resources.” | |
|  | Advantages over Medication | 2 (20.0%) | 3 (20.0%) | 5 (20.0%) | "Sometimes our clients don't want medication for sleep because it sedates them, things like that.” | |
|  | More Organized | 4 (40.0%) | 8 (53.3%) | 12 (48.0%) | “What the Berkeley program has done is it has given structure to help people develop good sleep." | |
|  | More Helpful | 1 (10.0%) | 5 (33.3%) | 6 (24.0%) | "I was in an inpatient psychiatric hospital...in that setting, I was using, oh, just, you know, just kind of casual, informal, in-the-moment. You know, I did do some groups. I did do some sleep hygiene groups and a lot of discussion around that. And I think, I think this sleep coaching is more effective..." | |
|  | Complementary | 1 (10.0%) | 2 (13.3%) | 3 (12.0%) | "I think that this has been kind of complementary, and given me more information" | |
|  | More Client-Centered | 0 (0.0%) | 4 (26.7%) | 4 (16.0%) | "...this is a much kinder, gentler way to get the same results in a way, that felt more, more client-centered and less risky, I think." | |
|  | Less Stigma | 1 (10.0%) | 2 (13.3%) | 3 (12.0%) | "It sort of like takes any kind of like stigma away, because it's like an area that we all have to focus on and work on is sleep." | |
| **Not Acceptable** |  |  |  |  |  | |
|  | Overly Simplified | 6 (60.0%) | 11 (73.3%) | 17 (68.0%) | "It’s not real detail-oriented...I understand that was to make it more accessible for a large range of folks, but definitely including options for...people who want to know more about the science..." | |
|  | Too Fast-Paced | 6 (60.0%) | 4 (26.7%) | 10 (40.0%) | “I think just going slower and focusing on one thing at a time...making it smaller, maybe like smaller increments of it.” | |
|  | Too Structured | 1 (10.0%) | 0 (0.0%) | 1 (4.0%) | “And [TranS-C] can be a little bit too routine based.” | |
|  | Clients Disliked Written Materials and Worksheets | 1 (10.0%) | 5 (33.3%) | 6 (24.0%) | "I guess some of the feedback is, I guess the client workbooks are in black and white, and so, and I would like tell them like, 'Oh, you can tear out, you know, this reminder of the rise up routine.' They were like, 'Oh, yeah, I guess.'" | |
|  | Suggestions to Improve Flexibility | 2 (20.0%) | 1 (6.7%) | 3 (12.0%) | "Being able to alter the sleep diary template on our end would be really helpful, because I found that adding a certain metrics on there was helpful for different participants." | |
|  | Similar to Other Approaches | 1 (10.0%) | 3 (20.0%) | 4 (16.0%) | "Yeah, I would, I have to say there were some similarities. So I’m a DBT focused provider in addition to CBT, which it seems a lot of CBT in the sleep treatment is used." | |
| **Appropriate** |  |  |  |  |  | |
|  | Appropriate Based on Client Characteristics | 0 (0.0%) | 4 (26.7%) | 4 (16.0%) | "I have very good, good, solid therapeutic alliance with [existing clients]...They have a little bit more buy in." | |
|  | Appropriate Based on Client Symptoms | 2 (20.0%) | 3 (20.0%) | 5 (20.0%) | "And then trauma, you know how trauma impacts sleep and how that can lead to severe mental illness and how that impacts sleep." | |
|  | Empowering | 2 (20.0%) | 3 (20.0%) | 5 (20.0%) | "The sleep treatment, when I got that from UC Berkeley, it really empowered me as a provider to work with my clients to expect more from their sleep and not to just go that, it is what it is, I only sleep for an hour or two and that’s just how it is." | |
|  | Sleep Is Important Topic | 9 (90.0%) | 11 (73.3%) | 20 (80.0%) | "And I do see with my clients when they have improved sleep, so many other things improve for them. Their mood, their energy levels, their concentration, their focus." | |
|  | Helps Clients | 8 (80.0%) | 11 (73.3%) | 19 (76.0%) | “Extremely [effective]. It’s been, for several clients, life altering.” | |
|  | Appropriate for Sleep Issues | 8 (80.0%) | 11 (73.3%) | 19 (76.0%) | "Initially, like when I started, I was like, you know, basically any time a client brought up some sleep issues, I’d tell them about the sleep study, the sleep program, and made an, offer to them and most clients, most were interested.” | |
|  | Fits with Professional Role | 0 (0.0%) | 2 (13.3%) | 2 (8.0%) | “The sleep coaching slash sleep treatment just was a natural fit for what I already do here because of my focus on everyday living skills and, you know, a client's ability to function.” | |
|  | Beneficial For Any Client Presentation | 4 (40.0%) | 11 (73.3%) | 15 (60.0%) | “Because it's just a human...need like...Regardless of what is going on with you physically or mentally, it's just an area of life that's universal.” | |
| **Not Appropriate** |  |  |  |  |  | |
|  | Not Appropriate Based on Client Characteristics | 8 (80.0%) | 13 (86.7%) | 21 (84.0%) | “Some of my clients were just not motivated to keep going with the treatment.” | |
|  | Not Appropriate Based on Client Symptoms | 6 (60.0%) | 5 (33.3%) | 11 (44.0%) | “If someone is, you know, decompensating into psychosis, you know, they're not to be able to, be able to think through and understand what I'm trying to articulate with them. And so, you know, when it comes to those pieces, I'm going to back off and just get to the very base stuff just to help them get through that moment. But once they're at a baseline where they can understand the material then we will go there and get back to it.” | |
|  | More Content/Sessions Needed | 8 (80.0%) | 6 (40.0%) | 14 (56.0%) | “I think it just gives them more time in having that at the forefront and then it would be maybe easier to engage in or keep it in their … the front of their mind for six weeks, I think, would just be more beneficial than the four weeks.” | |
|  | Depends on Clients’ Presentation | 8 (80.0%) | 5 (33.3%) | 13 (52.0%) | “I think that [effectiveness], of course, that is just different per client.” | |
| **Feasible** |  |  |  |  |  | |
|  | Client Characteristics Improved Feasibility | 1 (10.0%) | 0 (0.0%) | 1 (4.0%) | “Interest or willingness. You know, if they weren't super excited, but they were willing, I would go for it.” | |
|  | Provider Characteristics Improved Feasibility | 2 (20.0%) | 2 (13.3%) | 4 (16.0%) | “I value it highly so I can always make it a priority.” | |
|  | Treatment Characteristics Improved Feasibility | 2 (20.0%) | 11 (73.3%) | 13 (52.0%) | “I think the biggest thing is that it was so simply worded it was really digestible for the clients, which also made it easy to present. So it is easy to retain, you know, go through and remember the material and then be able to present it in the session." | |
|  | Implementation Characteristics Improved Feasibility | 3 (30.0%) | 5 (33.3%) | 8 (32.0%) | “The program helped me out by keeping track for me, of like how many sessions I had with each client when they send the weekly thing to remind us to fill it out.” | |
|  | Context Improved Feasibility | 2 (20.0%) | 3 (20.0%) | 4 (16.0%) | “I mean having my organization be on board with it helped." | |
| **Not Feasible** |  |  |  |  |  | |
|  | Client Characteristics Decreased Feasibility | 8 (80.0%) | 8 (53.3%) | 16 (64.0%) | “But some of the clients I'm already meeting with every other week or weekly, usually there's a lot going on. So I mean, I could feasibly see incorporating some time, but not a full session in addition to what we're already doing. If I'm already meeting them weekly or biweekly it’s because those are kind of my more intensive clients.” | |
|  | Treatment Characteristics Decreased Feasibility | 7 (70.0%) | 11 (73.3%) | 18 (72.0%) | “My appointments are about 45 minutes long with each client, so sometimes the time restraint was a little difficult to manage.” | |
|  | Context Decreased Feasibility | 4 (40.0%) | 3 (20.0%) | 7 (28.0%) | "And, yeah, workload. There are a lot of other things I have to do, of course, with these assessments and treatment plans and such, and I am juggling a lot of that. So that's part of the reason I haven't done as much as I want to.” | |
| **High Fidelity** |  |  |  |  |  | |
|  | Attended Supervision | 0 (0.0%) | 5 (33.3%) | 5 (20.0%) | "I also utilized the, can’t remember what it's called...it’s like a support group, but like the extra assistance provided during the week on the group call." | |
|  | Used Manual and Workbook | 9 (90.0%) | 11 (73.3%) | 20 (80.0%) | "I just used the book really." | |
|  | Did Not Make Any Changes | 0 (0.0%) | 1 (6.7%) | 1 (4.0%) | "I don’t think I implemented or changed anything." | |
|  | Dedicated Full Sessions to Treatment | 4 (40.0%) | 8 (53.3%) | 12 (48.0%) | "We did dedicate a couple of sessions just solely for sleep mainly and for going through the curriculum in the workbook together." | |
| **Low Fidelity** |  |  |  |  |  | |
|  | Inconsistent Supervision Attendance | 2 (20.0%) | 2 (13.3%) | 4 (16.0%) | "A couple of times where I wanted to do a drop-in group and then I wasn’t able to make it." | |
|  | Treatment Not Delivered Over Consecutive Weeks | 1 (10.0%) | 1 (6.7%) | 2 (8.0%) | "I would either be doing the sleep therapy for the entire session, or I will pull back completely." | |
|  | Not Full Use of Provider Manual and Client Workbook | 0 (0.0%) | 6 (40.0%) | 6 (24.0%) | "We used the workbook kind of as a jumping off point for our discussions... And then kind of just like I said, use the materials not so much as like a homework assignment or schoolwork, but more like an outline that I could go through and just kind of tick off items and make sure that I had shared the information that needed to be shared or that I thought would be helpful for the client." | |
|  | Used More Time Than Allotted | 9 (90.0%) | 12 (80.0%) | 21 (84.0%) | "I think [each module] took multiple sessions." “...really taking some intentional time to hit that for multiple sessions." | |
|  | Integrated Treatment with Other Session Content | 7 (70.0%) | 11 (73.3%) | 18 (72.0%) | "It’s really kind of embedded with some of the clinical goals...it’s been a little less structured... I can just integrate into the session as we’re moving along, we can point out different things about sleep that are relevant to how they might be feeling." | |
|  | Integrated Additional Materials on Sleep | 6 (60.0%) | 6 (40.0%) | 12 (48.0%) | "I’ve been talking about this dream completion technique, which I learned about." | |
|  | Skipped Materials | 8 (80.0%) | 5 (33.3%) | 13 (52.0%) | "I would skip things not because I didn't think it was helpful, but because it was something the patient already knew. Like patients who just generally know that they shouldn't be on their screens or TV before bedtime. We would skip over those sections or about drinking coffee too late." | |
|  | Stopped TranS-C | 2 (20.0%) | 1 (6.7%) | 3 (12.0%) | "Sometimes we start and we don’t finish." | |
|  | Modified Delivery | 4 (40.0%) | 10 (66.7%) | 14 (56.0%) | "Sometimes I would talk about my own experiences and what, you know, I've taken out of the training and been like, 'Well, this one really seemed to help me, I wonder if there's one that would help you.'" | |
| **Influences on Fidelity** | |  |  | | |  |
|  | More Time Needed | 5 (50.0%) | 5 (33.3%) | 10 (40.0%) | "I think the biggest thing was the modules might be a lot to cover in one day or even a few sessions." | |
|  | Client Interest and Motivation | 5 (50.0%) | 9 (60.0%) | 14 (56.0%) | "You know, some, some patients...we would spend the entirety of the session on the sleep treatment and that worked for them. They were interested in it. And that's how it went. Other clients became less interested as time went on, and they, they wanted to use some of the session for, like other therapeutic work as well." | |
|  | Client Clinical Presentation | 8 (80.0%) | 11 (73.3%) | 19 (76.0%) | "You know, I’ve done bits of sleep treatment with a couple of clients who were unhoused and that there I was definitely shifting the tips and the suggestions to things they could control. You know, let's brainstorm a safer place for you to sleep where there's less noise." | |
|  | Supervision Group Was Inconsistent | 1 (10.0%) | 0 (0.0%) | 1 (4.0%) | "And then there's a couple of times it’s happens, where it gets rescheduled last minute and then that has made it has made me hesitant to commit." | |
|  | Provider Characteristics | 0 (0.0%) | 4 (26.7%) | 4 (16.0%) | "I encouraged us focusing on it for the whole session or sort of big chunk because I thought it was important." | |
| **Sustainable** |  |  |  |  |  | |
|  | Importance Of Treating Sleep | 1 (10.0%) | 4 (26.7%) | 5 (20.0%) | "I do think sleep is important." | |
|  | Relevant To Clients | 8 (80.0%) | 3 (20.0%) | 11 (44.0%) | "Yes I will [continue to use TranS-C]...because most of my clients do come in with something that has to do with sleep." | |
|  | Helpful For Clients | 6 (60.0%) | 8 (53.3%) | 14 (56.0%) | "Well, because they're getting good results, you know, really good results. You know, getting better sleep, fewer psychotic symptoms, less relapse." | |
|  | Feasible | 1 (10.0%) | 3 (20.0%) | 4 (16.0%) | "I think everything in the manual, it's pretty simple to implement. So that helps, because they're able to actually do it without too much effort. So I think, I think I will [continue to use it]." | |
|  | Sustainable Based on Treatment Characteristics | 3 (30.0%) | 10 (66.7%) | 13 (52.0%) | "Having this curriculum to kind of draw upon gives me more of a formalized resource to kind of go over with clients and help teach them things that they can do to manage their sleep in such a way that their psychiatric symptoms are under better control." | |
|  | Gain Proficiency | 1 (10.0%) | 1 (6.7%) | 2 (8.0%) | "I want to become more comfortable with [the sleep treatment]. I want it to be more seamless, like scope of practice for myself." | |
|  | Implementation Characteristics | 2 (20.0%) | 7 (46.7%) | 9 (36.0%) | "I think it’s just getting that normal refresher every once in a while and email to sort of incentivize the clinician side of things." | |
| **Not Sustainable** |  |  |  |  |  | |
|  | Not Sustainable Based on Treatment Characteristics | 2 (20.0%) | 4 (26.7%) | 6 (24.0%) | "Clients do kind of have a hard time working with a book, like locating a book, like, where's my book?" | |
|  | Not Sustainable Due to Contextual Factors | 2 (20.0%) | 4 (26.7%) | 6 (24.0%) | "We want to break that resistance that when people hear, because right now in our work environment, like if someone hears that they have to be trained to do this, they immediately go, it's like, I can't handle any more work." | |
|  | Need Access to More Resources | 3 (30.0%) | 7 (46.7%) | 10 (40.0%) | "There's so much out there and there's no way I can get all that at just a manual." | |
|  | Will Modify Moving Forward | 2 (20.0%) | 7 (46.7%) | 9 (36.0%) | "I think I would combine my own approach with the sleep treatment." | |

*Note*. Standard = Standard TranS-C. Adapted = Adapted TranS-C. TranS-C = Transdiagnostic Intervention for Sleep and Circadian Dysfunction. SMI = serious mental illness. Providers used terms “sleep treatment” and “sleep coaching” to refer to TranS-C. Brackets and ellipses are used for clarification. The percentages indicate the proportion of providers who had a response coded with a given theme, by condition.

Semi-Structured Interview Questions

1. **Fidelity:** We’d like to start by hearing about the “nuts and bolts” of how you integrated the sleep treatment in your work with your clients. Here, we’re interested in learning about the logistical approach or approaches that you used to deliver the sleep treatment. For example, some providers discuss tips from the sleep treatment for a few minutes at the end of a session, whereas others focus on sleep treatment exclusively for entire sessions over several weeks. Did you use the therapist manual and client workbook? Or perhaps you used other approaches? Please describe the approach or approaches that you used to deliver the sleep treatment.
2. **Feasibility:** Did the sleep treatment, as you just described using it, seem feasible or doable to use routinely with your clients, given your workload, session time, and clinical priorities with your clients? Why or why not?
3. **Acceptability:** In mental health care, we’re only just starting to understand and emphasize sleep in mental health treatment, as you probably know. Some providers have developed their own ways of addressing sleep problems with your clients. If you’ve addressed sleep with your clients outside the UC Berkeley sleep treatment, how does the UC Berkeley sleep treatment compare to other approaches that you’ve used, in terms of how effective it was?
4. **Appropriateness:** This sleep treatment was developed to be transdiagnostic, meaning that it was developed to be used with people who have a wide range of sleep and mental health problems. Thinking about the clients you’ve used the sleep treatment with—or hypothetically about different clients on your caseload—does the sleep treatment seem useful across clients with different symptoms or problems?
5. **Appropriateness:** As a follow-up: Our sleep treatment was also developed to treat people who have experienced severe and impairing mental illness symptoms for at least a year. Do you have any suggestions for changing the treatment to better treat sleep problems for clients who meet these criteria?
6. **Adoption:** Shifting gears a little bit, why did you initially decide to become a sleep therapist with the study?
7. **Adoption:** What guided your decision to use the sleep treatment with certain clients and not others? *If needed for follow-up*: What guided your decisions on when NOT to use the sleep treatment with certain clients?
8. **Fidelity:** Beyond what you described in the first question about the “nuts and bolts” of administering the sleep treatment, did you personalize or make any other changes to the sleep treatment to fit better with your schedule or your clients’ needs? Here, we’re trying to understand any changes you made and why, which might give us ideas for other ways to improve the sleep treatment moving forward. And I can provide some examples if that would be helpful.

*If requests examples:* As some other examples, did you spend more time on some modules than others based on different clients’needs? Did you personalize any examples or tips for particular clients? Did you add anything that you’ve learned in other trainings or your own flavor? Did you skip anything that you didn’t think would be helpful for that particular client? Please describe any changes you made and why.

1. **Sustainability:** Do you think that you will continue to use the sleep treatment with your clients in the future? We’d love to know why or why not?
2. **Sustainability:** What would make it easier for you to continue to use the sleep treatment in the future?

*If needed for follow-up:* As some examples, this could be related to the treatment itself, the trainings, supervision, consultation groups with other clinicians, support or incentives from leadership, streamlining the referral process – so here we’re really wondering about anything that could make it easier for you.

1. **Wrap-Up:** Is anything else that you’d like to share with us about your experience as a sleep therapist or using the sleep treatment that we haven’t covered yet?
